# Retraction Note: Arsenic album 30C exhibits crystalline nano structure of arsenic trioxide and modulates innate immune markers in murine macrophage cell lines

**DOI:** 10.1038/s41598-024-70287-9

**Published:** 2024-08-21

**Authors:** Suvasmita Rath, Jyoti Prava Jema, Kamali Kesavan, Sagar Mallick, Jyotsnarani Pradhan, Gagan Bihari Nityananda Chainy, Debadatta Nayak, Subhash Kaushik, Jagneshwar Dandapat

**Affiliations:** 1 Centre of Environment, Climate Change and Public Health, Utkal University, Vani Vihar, Bhubaneswar, Odisha India; 2 Post Graduate Department of Biotechnology, Utkal University, Bhubaneswar, Odisha India; 3 CSIR-Institute of Minerals and Materials Technology, Bhubaneswar, Odisha India; 4 Central Council for Research in Homeopathy, New Delhi, India; 5 Centre of Excellence in Integrated Omics and Computational Biology, Utkal University, Bhubaneswar, Odisha 751004 India

Retraction of: *Scientific Reports* 10.1038/s41598-024-51319-w, published online 07 January 2024

The Editors have retracted this Article.

After publication, concerns were raised about the reagents used in this study, in particular that the arsenic trioxide solution is diluted beyond the point at which any active molecules are expected to be present. Post-publication peer review confirmed that the nature of the particles detected in the study is unclear. This means that without further corroborative evidence, the data presented in the paper are not sufficient to attribute the effects observed after treating cells with the compound. The Editors therefore no longer have confidence in the results reported in this Article.

None of the authors have responded to correspondence from the Editors about this retraction.

